# Meconium Transferrin and Ferritin as Markers of Homeostasis in the Developing Fetus

**DOI:** 10.3390/ijms242115937

**Published:** 2023-11-03

**Authors:** Ewa Skarżyńska, Klaudia Mularczyk, Tadeusz Issat, Artur Jakimiuk, Barbara Lisowska-Myjak

**Affiliations:** 1 Department of Laboratory Medicine, Medical University of Warsaw, 02-097 Warsaw, Poland; klaudia.mularczyk99@gmail.com; 2 Department of Obstetrics and Gynecology, Institute of Mother and Child, 01-211 Warsaw, Poland; tadeusz.issat@imid.med.pl; 3 Department of Obstetrics, Women’s Diseases and Gynecologic Oncology, National Medical Institute of the Ministry of the Interior and Administration, 02-507 Warsaw, Poland; jakimiuk@yahoo.com; 4 Center for Reproductive Health, Institute of Mother and Child, 01-211 Warsaw, Poland; 5 Department of Biochemistry and Pharmacogenomics, Medical University of Warsaw, 02-097 Warsaw, Poland

**Keywords:** meconium, birth weight, ferritin, transferrin, intrauterine environment

## Abstract

The molecular mechanisms regulating homeostasis in the developing fetus have not been satisfactorily elucidated. Meconium contains substances accumulated in the fetal intestines. Measurements of transferrin and ferritin concentrations in meconium and assessment of transferrin–ferritin relationships could enhance knowledge about specific processes of the intrauterine period involving the two proteins and their effects on the development and growth of the fetus. Transferrin and ferritin concentrations were measured by ELISA in the homogenates of first meconium portions from 125 neonates. Higher birth weight was associated with lower ferritin concentrations in meconium (r = −0.22, *p* = 0.015). In neonates with a birth weight of more than 3750 g, there was a positive correlation between transferrin and ferritin concentrations (r = 0.51, *p* = 0.003). With meconium transferrin concentrations above 43.52 µg/g, a negative correlation between transferrin and ferritin was established (r = −0.37, *p* = 0.036), while with transferrin concentrations below 43.52 µg/g, the correlations between the birth weight and the meconium transferrin and ferritin concentrations were negative (r = −0.61, *p* < 0.001 and r = −0.43, *p* = 0.017, respectively). Measurements of transferrin and ferritin in meconium specimens create a new use for these common biomarkers to improve our understanding of the effects of homeostasis in utero on the fetal development and growth. Establishing reference ranges of meconium transferrin and ferritin concentrations and their association with the clinical parameters during pregnancy could aid in the assessment of the impact of intrauterine life on the health status of the neonate and its adaptation to extrauterine life.

## 1. Introduction

Clinicians emphasize the need for better, evidence-informed understanding of the impact of the intrauterine environment on neonates’ adaptation to extrauterine life and their further development [1,2,3]. Specific, non-invasively obtained clinical specimens for biomarker identification and analysis are required, but at present, their apparent scarcity considerably limits the use of clinical chemistry to assess fetal development and fetal growth.

The molecular mechanisms regulating the supply to the fetus of components required for its development and growth remain to be elucidated. Iron acquired in the fetal period is stored to meet iron requirements in the first 4 to 6 months after birth [4,5,6]. Our choice of transferrin and ferritin as biomarkers of fetal development in utero was based on the established diagnostic use of serum levels of transferrin and ferritin, which are both known to participate in iron transport and storage [7,8]. The complex regulation of iron metabolism in humans has been well researched, including more recent data on the role of transmembrane transporters of iron [8,9,10,11], but it is not clear whether in the intrauterine environment transferrin and ferritin have a similar function in maintaining normal development and growth of the fetus.

Meconium is proposed as a readily available and non-invasively obtainable biological material formed during the fetal period which remains in the intestines of the developing fetus to be passed only after birth [12,13]. The presence of components accumulating in meconium from week 12 confirms that they are in direct contact with the fetus, while their properties and concentrations may provide information about their actual biological role(s) in the intrauterine environment [13]. The possible sources of these meconium components are bile, amniotic fluid swallowed by the fetus or fetal urine [13]. The presence of transferrin and ferritin in meconium has been confirmed by earlier proteomic studies [12].

The aim of this study was to measure transferrin and ferritin concentrations in meconium specimens and assess their possible correlation with birth weight in study neonates.

## 2. Results

Table 1 shows transferrin and ferritin concentrations measured in the same meconium specimens from 125 neonates. The birth weights (g) ranged from 1860 to 4960, mean ± SD: 3474 ± 483, median 3520.

A range of 600-fold for transferrin concentrations (µg/g) was calculated compared to a range of 20-fold for ferritin, which is confirmed by a coefficient of variation (CV) for transferrin, which is over three times as much as the coefficient of variation for ferritin and a large dispersion of the T/F ratio, which indicates the relationship between transferrin and ferritin in meconium.

**Figure 1:**
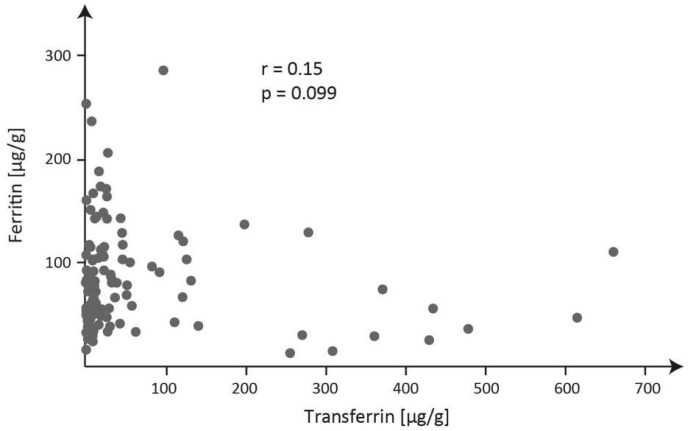
The correlation between the concentrations of transferrin and ferritin in meconium.

The graph in Figure 1 examines correlations between the concentrations of transferrin and ferritin in 125 meconium specimens.

Table 2 presents increasing transferrin concentrations in meconium separated intro quartiles and the corresponding values of meconium ferritin concentrations, T/F ratio and birth weight across the four quartiles.

Neonates in the fourth quartile had meconium transferrin concentrations exceeding 43.52 µg/g. Ferritin concentrations gradually increased with the increasing transferrin concentrations, although the differences in ferritin concentrations between quartiles (*p* = 0.016) were less dynamic than in the case of transferrin. The T/F ratio which reflects the differences between the concentrations of transferrin and ferritin increases across quartiles especially with meconium transferrin concentrations exceeding 43.52 µg/g.

The relationship between birth weight and both meconium transferrin (r = −0.16, *p* = 0.070) and meconium ferritin (r = −0.22, *p* = 0.015) was negative, but the correlation was statistically significant for ferritin only.

Table 3 examines the correlations between meconium transferrin and ferritin concentrations and between their concentrations and T/F ratio and birth weight across the transferrin quartiles.

The associations between the meconium transferrin concentrations over quartiles and the corresponding ferritin concentrations, T/F ratio and birth weight vary. Statistically significant correlations between transferrin and ferritin concentrations, positive (r = 0.43) and negative (r = −0.37), were observed in the second and fourth transferrin quartiles, respectively. This negative correlation was established in 25% of neonates with the meconium transferrin concentrations over 43.52 µg/g. There was also a negative correlation between the birth weight and the concentrations of transferrin (r = −0.61) and ferritin (r = −0.43) seen in the second and third transferrin quartiles, respectively.

Table 4 presents the neonates separated into four quartiles by increasing birth weight and the corresponding meconium concentrations of transferrin and ferritin and the T/F ratio.

The results demonstrated that the meconium transferrin and ferritin concentrations tended to decrease with the increasing birth weight, although they were statistically significant for ferritin only (*p* = 0.008), whose concentrations were significantly the highest in the first (0–25%) compared to the lowest concentrations in the fourth (75–100%) quartile of birth weight. The lack of statistical significance for transferrin concentrations in meconium was due to the large scatter of results. The T/F ratio which reflects the relative proportions of the two proteins in meconium did not show any significant differences (*p* > 0.05). Interestingly, the dispersion of the T/F ratio remained large over the birth weight quartiles.

**Figure 2:**
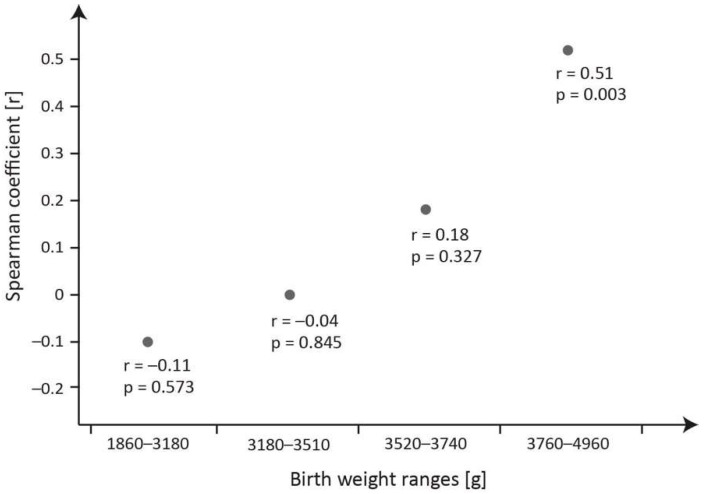
The relationship between the meconium transferrin and ferritin concentrations across birth weight quartiles.

Figure 2 presents the Spearman’s coefficients (r) for the concentrations of transferrin vs. ferritin across birth weight quartiles.

Figure 2 shows changes in the Spearman’s correlation coefficients (r) for meconium transferrin vs. ferritin across birth weight quartiles. A dynamic growth tendency is seen in a significant positive correlation between the two proteins (r = 0.51) in neonates with a birth weight exceeding 3760 g.

## 3. Discussion

The results suggest new ways for acquiring knowledge about the intrauterine environment in which a fetus develops by determining the meconium concentrations of proteins with known biological functions.

The practical objective of this study was to assess the relationship between transferrin and ferritin concentrations in meconium as potential biomarkers of fetal environment with the birth weight. The finding of decreased concentrations of the two proteins in meconium corresponding to the increased birth weight may confirm the observations by other authors [14] of a negative association between total protein concentrations in amniotic fluid and infant birth weight. In their opinion, proteins swallowed by a fetus with amniotic fluid are an important source of nutrients for its development and growth. Considering that proteins found in meconium are provided with amniotic fluid [12], low concentrations of transferrin and ferritin may confirm their involvement in increasing birth weight as evidenced by a significant negative correlation between the birth weight in the study group and the meconium ferritin concentrations. A question arises as to whether the cut-off values for meconium transferrin and ferritin when determined could be used as biomarkers for an adequate nutritional status of the fetus promoting its optimal intrauterine development.

Although the meconium concentrations of transferrin and ferritin tended to decrease in line with increases in birth weight, no significant correlation was established between the two proteins in the entire study group. Considering a scatter of 600-fold for transferrin concentration, the cut-off of 43.52 µg/g of transferrin in 75% of neonates was below that value. Additionally, in neonates with a birth weight below 3760 g, no significant correlations between transferrin and ferritin in meconium were established (Figure 2) in contrast to neonates with a birth weight above 3760 g, where the correlation was significant. Differences in the correlations between transferrin and ferritin in meconium and additionally their relationships with the birth weight (Table 3) allow posing a question regarding whether the properties of the two proteins specific to the intrauterine environment may determine their effect on gaining birth weight.

Transferrin and ferritin are proteins which utilize different mechanisms including those related to gender [15] to achieve a common goal, i.e., regulation of adequate iron supply and homeostasis, but also protection of the body against toxic effects of free iron. Ferritin is the iron-storage protein responsible for keeping an adequate amount of iron in the body, while transferrin with its iron-binding capacity transports iron to the target tissues [1,16,17].

The biological role of a 600-fold scatter of transferrin concentrations compared a 20-fold scatter for ferritin remains unclear. Apart from the initial stage of iron transfer from the maternal circulation to the fetus, our understanding of how iron is transported across the placenta to the fetus and what transporter proteins and mechanisms are involved in the regulation of further iron distribution in the intrauterine environment is limited [6,11,18]. The results we present demonstrate that despite differences in the birth weights among the neonates included in the study, there were no significant differences in the T/F ratio reflecting the proportions of transferrin and ferritin in meconium, although their considerable variability suggests some additional, individual mechanisms regulating their concentrations. The lack of any correlation between the concentrations of transferrin and ferritin may confirm the hypothesis of a protein biosynthesis pathway specific to the period in utero [19]. Amniotic fluid transferrin differs in structure and function from serum transferrin. It has been suggested that the trophoblast is involved in the regulation of amniotic fluid transferrin concentrations, and the particular biological role of amniotic fluid transferrin is to block E-selectin-mediated cell adhesion [19]. It seems that the evaluation of meconium transferrin should not just rely on the detection of its decreased level and saturation, which is of recognized diagnostic significance in tests on serum samples, but also include the interpretation of elevated concentrations. The complex, bidirectional mechanism of transferrin’s participation in processes related to maintaining iron homeostasis and immunological balance in the environment of the developing fetus requires further research and explanation.

In clinical practice, the iron status of a neonate is not routinely assessed, as it is assumed that the supply of iron from the mother to the fetus remains adequate even when the mother suffers from mild to moderate iron deficiency anemia [5,16,20]. Little is known about anemia and iron status in neonates, as children under one year of age are not routinely screened for anemia [20]. The results we present indicate the need for further investigations to elucidate the specific role(s) of the iron regulatory proteins and their adjustment to the local intrauterine mechanisms of iron supply to the fetus as well as their involvement in the regulation of processes of transition from intrauterine to extrauterine life.

The main limitation of our study is the lack of parallel determinations of transferrin and ferritin concentrations in the pregnant woman and her newborn, which could help assess the iron status and participation of these proteins in maternal–fetal transport. The mechanisms of transport of these proteins from the future mother to the fetus and their origin in meconium are unclear. Several studies have demonstrated the usefulness of measuring ferritin concentrations in pregnant women’s serum as a factor in predicting anemia [21,22]; therefore, a comparison of these results with the determination of ferritin in newborn meconium may provide additional knowledge in explaining the connections between mother and fetus.

## 4. Materials and Methods

### 4.1. Subjects

The study included 125 neonates born in the Department of Obstetrics, Women’s Diseases and Gynecologic Oncology, National Medical Institute of the Ministry of the Interior and Administration in Warsaw.

Parents/guardians of all participating neonates signed an informed consent form after receiving an explanation of the study objectives and methodology.

### 4.2. Material

First meconium only released prior to birth during labor or directly after birth was used in the study. The specimen was transferred using a spatula into a 50 mL plastic tube and frozen at –20 °C for up to 7 days, and next, a meconium homogenate was prepared.

The empty tubes were weighed prior to adding meconium and reweighed after filling. The date, time and weight of each meconium collection were recorded. The weight of a meconium specimen (g) ranged from 0.484 to 9.899, mean ± SD: 5.044 ± 2.473, median 5.168.

To prepare the homogenate, PBS (phosphate-buffered saline pH 7.4) was added to a tube with meconium: one part by weight of meconium per four parts by weight of PBS added in two equal portions. After the first PBS portion was added, the tube was placed on a hematology mixer for 15 min and then shaken in a horizontal position using an LP300Hk shaker for 1 h. After adding the second PBS portion, the procedure was repeated. The homogenate was transferred to Eppendorf tubes and stored at −80 °C. Prior to protein measurements, the homogenates were thawed for 24 h in a refrigerator. Next, they were mixed on a hematology mixer at a room temperature for one hour.

### 4.3. Methods

Transferrin and ferritin concentrations in meconium were measured by ELISA using Assaypro LLC ELISA kits (3400 Harry S Truman Blvd, St. Charles, MO 63301, USA, www.assaypro.com, (accessed on 1 January 2020)) according to the manufacturer`s instructions:

1. AssayMaxTM Human Ferritin ELISA Kit (Catalog Number: EF2003-1);
2. AssayMaxTM Human Transferrin ELISA Kit (Catalog Number: ET3105-1).

Measurements were performed in accordance with the principles of Good Laboratory Practice (GLP) in duplicate, and then the mean value was calculated and reported as a final concentration.

### 4.4. Statistical Analysis

Statistical analysis using Statistica Version 13 (StatSoft Inc., TIBCO Software Inc., Palo Alto, California, USA) was performed. In the first stage, the normality of variables was assessed by the Shapiro–Wilk test (all samples *n* = 125, in consecutive quartiles *n* = 31, *n* = 31, *n* = 31, *n* = 32, respectively, relative to increases in transferrin concentrations and increases in birth weight). The Kruskal–Wallis ANOVA test and the median test were used for comparisons; when *p* < 0.05, multiple comparisons among means were performed to determine the difference in a specific variable between quartiles. The Spearman’s correlation coefficient (r) was calculated for assessing correlations between variables. The statistical significance level was assumed as *p* < 0.05 [23].

## 5. Conclusions

In conclusion, determining transferrin and ferritin concentrations in meconium opens a perspective for the elucidation of their role in maintaining homeostasis during fetal development in utero. The relationship between the meconium transferrin and ferritin concentrations and the birth weight found in this study suggests the influence of the two proteins on the intrauterine conditions in which a fetus develops. Measurements of transferrin and ferritin concentrations in meconium could be of key importance for the assessment of homeostasis and equip the newborn in iron as well as serve as early postnatal predictors of possible developmental disorders in the following months and years of life. However, in order to confirm the rationale of the study and our conclusions, further research should include studies comparing the concentrations of transferrin and ferritin in the serum and meconium of the newborn, and additionally, researchers should carry out a long-term assessment of the concentrations of these proteins in comparison with those obtained in meconium.

## Figures and Tables

**Table 1:** Meconium concentrations of transferrin and ferritin and transferrin to ferritin ratio.

Variable	Mean ± SD	Median	Range	CV *
Transferrin (µg/g)	59.98 ± 118.67	13.06	1.06–655.74	197.9
Ferritin (µg/g)	79.72 ± 50.40	66.61	13.01–286.25	63.2
T/F **	1.34 ± 3.59	0.21	0.01–20.53	268.3

* CV = coefficient of variation (%), ** T/F = transferrin to ferritin ratio in the same meconium specimen.

**Table 2:** Meconium transferrin concentrations separated into four quartiles and the corresponding meconium ferritin concentrations, T/F ratio and birth weight.

Variable	0–25%	25–50%	50–75%	75–100%	
	*n* = 31	*n* = 31	*n* = 31	*n* = 32	*p*
Transferrin (µg/g)	4.31 ± 2.30 ^a^	10.13 ± 1.32 ^b^	24.52 ± 7.31 ^c^	196.55 ± 173.71 ^d^	0.000 *
	3.94	10.17	23.73	121.57	
	1.06–7.50	7.50–12.62	13.06–39.74	43.52–655.74	
Ferritin (µg/g)	71.98 ± 48.91	66.76 ± 46.32 ^a^	97.59 ± 48.70 ^b^	82.46 ± 54.01	0.016 **
	54.01	53.58	86.60	76.61	
	16.43–254.03	24.25–237.03	33.70–206.51	13.01–286.25	
T/F ratio	0.08 ± 0.06 ^a^	0.20 ± 0.09 ^b^	0.32 ± 0.20 ^c^	4.64 ± 6.03 ^d^	0.000 ***
	0.08	0.19	0.26	1.52	
	0.01–0.21	0.04–0.41	0.09–0.84	0.31–20.53	
Birth weight (g)	3447 ± 379	3759 ± 467 ^a^	3385 ± 423 ^b^	3308 ± 540 ^c^	0.005 ****
	3570	3720	3450	3310	
	2700–4070	2820–4960	1860–4080	1940–4150	

The measurements are presented as mean ± SD; median; range; *p* value for multiple comparisons (two-way) in the Kruskal–Wallis test. Statistically significant differences: * a vs. b, c, d; b vs. c, d; c vs. d, ** a vs. b, *** a vs. b, c, d; b vs. d; c vs. d, **** a vs. b, c.

**Table 3:** The effect of increasing meconium transferrin concentrations on the relationships of transferrin vs. ferritin and of transferrin, ferritin and T/F ratio vs. birth weight.

Pair of Variables	Spearman’s Correlation Coefficients (r) Across Meconium Transferrin Quartiles (Transferrin Concentration in µg/g)			
	0–25%	25–50%	50–75%	75–100%
	*n* = 31	*n* = 31	*n* = 31	*n* = 32
	(1.06–7.50)	(7.50–12.62)	(13.06–39.74)	(43.52–655.74)
Transferrin vs. ferritin	r = −0.02	**r = 0.43**	r = −0.04	**r = −0.37**
	*p* = 0.924	***p*** **= 0.016**	*p* = 0.839	***p*** **= 0.036**
Birth weight vs. transferrin	r = 0.05	**r = −0.61**	r = 0.15	r = 0.19
	*p* = 0.789	***p*** **< 0.001**	*p* = 0.422	*p* = 0.309
Birth weight vs. ferritin	r = −0.29	r = −0.31	**r = −0.43**	r = −0.01
	*p* = 0.105	*p* = 0.095	***p*** **= 0.017**	*p* = 0.949
Birth weight vs. T/F ratio	r = 0.17	r = 0.16	**r = 0.46**	r = 0.12
	*p* = 0.396	*p* = 0.380	***p*** **= 0.009**	*p* = 0.509

Statistically significant correlations between the variables (*p* < 0.05) in bold.

**Table 4:** Quartiles of birth weight and the corresponding meconium concentrations of transferrin and ferritin and the T/F ratio.

Variable	0–25%	25–50%	50–75%	75–100%	
	*n* = 31	*n* = 31	*n* = 31	*n* = 32	*p*
Birth weight (g)	2860 ± 338 ^a^	3338 ± 100 ^b^	3649 ± 61 ^c^	4031 ± 253 ^d^	0.000 *
	2960	3340	3670	3980	
	1860–3180	3185–3510	3520–3740	3760–4960	
Transferrin (µg/g)	67.22 ± 119.53	58.25 ± 109.12	76.02 ± 159.74	39.09 ± 74.29	0.517
	23.73	16.84	11.28	10.17	
	1.69–610.72	3.60–427.08	1.06–655.74	1.72–369.35	
Ferritin (µg/g)	104.73 ± 66.51 ^a^	66.65 ± 33.89	82.21 ± 40.51	65.74 ± 46.63 ^b^	0.008 **
	93.01	65.74	81.06	53.14	
	14.97–286.25	13.01–142.75	32.73–174.09	24.25–237.03	
T/F ratio	1.57 ± 4.24	2.09 ± 5.03	1.16 ± 2.79	0.55 ± 0.92	0.701
	0.25	0.20	0.17	0.28	
	0.01–20.53	0.08–19.58	0.01–13.09	0.04–4.96	

The measurements are presented as mean ± SD; median; range; *p* value for multiple comparisons (two-way) in the Kruskal–Wallis test. Statistically significant differences: * a vs. b, c, d; b vs. c, d; c vs. d, ** a vs. b.

## Data Availability

The data presented in this study are available on request from the corresponding authors. The data are not publicly available due to privacy.
